# Persistence of information flow: A multiscale characterization of human brain

**DOI:** 10.1162/netn_a_00203

**Published:** 2021-08-30

**Authors:** Barbara Benigni, Arsham Ghavasieh, Alessandra Corso, Valeria d’Andrea, Manlio De Domenico

**Affiliations:** Department of Information Engineering and Computer Science, University of Trento, Trento, Italy; CoMuNe Lab, Fondazione Bruno Kessler, Trento, Italy; CoMuNe Lab, Fondazione Bruno Kessler, Trento, Italy; Department of Physics, University of Trento, Trento, Italy; CoMuNe Lab, Fondazione Bruno Kessler, Trento, Italy; Department of Mathematics, University of Trento, Trento, Italy; CoMuNe Lab, Fondazione Bruno Kessler, Trento, Italy; CoMuNe Lab, Fondazione Bruno Kessler, Trento, Italy

**Keywords:** Network communication, Information flow, Spectral entropy, Alzheimer’s disease, Mild cognitive impairment

## Abstract

Information exchange in the human brain is crucial for vital tasks and to drive diseases. Neuroimaging techniques allow for the indirect measurement of information flows among brain areas and, consequently, for reconstructing connectomes analyzed through the lens of network science. However, standard analyses usually focus on a small set of network indicators and their joint probability distribution. Here, we propose an information-theoretic approach for the analysis of synthetic brain networks (based on generative models) and empirical brain networks, and to assess connectome’s information capacity at different stages of dementia. Remarkably, our framework accounts for the whole network state, overcoming limitations due to limited sets of descriptors, and is used to probe human connectomes at different scales. We find that the spectral entropy of empirical data lies between two generative models, indicating an interpolation between modular and geometry-driven structural features. In fact, we show that the mesoscale is suitable for characterizing the differences between brain networks and their generative models. Finally, from the analysis of connectomes obtained from healthy and unhealthy subjects, we demonstrate that significant differences between healthy individuals and the ones affected by Alzheimer’s disease arise at the microscale (max. posterior probability smaller than 1%) and at the mesoscale (max. posterior probability smaller than 10%).

## Author Summary

Capitalizing on network information theory we propose a new framework for the analysis and comparison of empirical and synthetic human connectomes, in health and disease. We quantitatively assess the ability of our approach in identifying differences and similarities in the structural connectivity of the human brain, ranging between microscopic and macroscopic scales. Relying on two different diffusion processes, namely classic and maximal entropy random walks, we show that the mesoscale is suitable for capturing differences between empirical connectomes and their synthetic models, while differences between healthy subjects and patients affected by Alzheimer’s disease are mostly significant at the microscopic scale.

## INTRODUCTION

The human brain is usually referred to as an emblematic example of efficient complex systems, where neurons (i.e., the units) process a huge amount of information by signaling through synaptic transmission (i.e., the links). To better understand how information flows within the brain, imaging techniques are widely used to infer structural and functional relationships between distinct areas of the brain obtained according to some parcellation in regions of interests (Assaf & Pasternak, 2007; van den Heuvel & Pol, 2010). The resulting maps coarse grain the brain into complex networks of much smaller size, typically of the order of a few hundreds or thousands of areas that are analyzed through the lens of network science (Bassett & Sporns, 2017; Bullmore & Sporns, 2009, 2012; Deco, Jirsa, & McIntosh, 2010; Deco, Tononi, Boly, & Kringelbach, 2015; Fox et al., 2005; Meunier, Lambiotte, Fornito, Ersche, & Bullmore, 2009; Papo, Buldú, Boccaletti, & Bullmore, 2014; Schirner, McIntosh, Jirsa, Deco, & Ritter, 2018; Yamamoto et al., 2018; Zalesky, Fornito, & Bullmore, 2010).

On the one hand, network descriptors are used for a variety of applications, from quantifying the characteristic geodesic distance among units to unravel their mesoscale organization into functional modules (Betzel, 2020; Sporns & Betzel, 2016), from identifying nodes central with respect to information exchange to more sophisticated roles such flow attraction and connector hubs (Bertolero, Yeo, Bassett, & D’Esposito, 2018; Bullmore & Sporns, 2012; Hwang, Hallquist, & Luna, 2012; Lohmann et al., 2010; van den Heuvel & Sporns, 2013), from both nodal and link perspectives (Faskowitz, Esfahlani, Jo, Sporns, & Betzel, 2020). Recently, more sophisticated models such as multilayer networks (De Domenico et al., 2013) have been used to this aim: in that case, the layers encode distinct types of relationships among units, either accounting for multimodal measurements (Battiston, Guillon, Chavez, Latora, & de Vico Fallani, 2018; Guillon et al., 2019) or for correlations in the temporal (Bassett et al., 2011) or frequency (Buldú & Porter, 2018; De Domenico, Sasai, & Arenas, 2016; Williamson, De Domenico, & Kadis, 2021) domains (see De Domenico, 2017, for a review).

Network science has proven to be a powerful analytical tool for unraveling the properties of the human brain, and several models have been proposed to reproduce the most widely observed salient features, from small-worldness (Bassett & Bullmore, 2016; Liao et al., 2011; Muldoon, Bridgeford, & Bassett, 2016), usually related to an amount of triadic closure higher than chance together with a characteristic geodesic length lower than random expectation (Watts & Strogatz, 1998), to modularity (Betzel, 2020; Sporns & Betzel, 2016), that is, the organization of units into groups (Fortunato, 2010) with a biological function, enhancing our understanding of brain structure and function in both health and disease (A. Alexander-Bloch et al., 2012; A. F. Alexander-Bloch et al., 2013; Amico & Goñi, 2018; Castellanos & Proal, 2012; De Domenico et al., 2016; de Vico Fallani, Richiardi, Chavez, & Achard, 2014; Fornito & Bullmore, 2015; Fornito, Zalesky, & Breakspear, 2015; Griffiths et al., 2021; Guillon et al., 2017; Hernandez, Rudie, Green, Bookheimer, & Dapretto, 2015; Lella & Estrada, 2020; Qiu et al., 2011; Rubinov & Bullmore, 2013; Rudie et al., 2013). For instance, it has been recently shown that interareal Euclidean distance plays an important role in shaping the structural organization of brain networks (Betzel & Bassett, 2018), opening the door to the exploration of models driven by geometry (see Betzel et al., 2016; Vértes et al., 2012, and references therein) and latent geometry (Allard & Serrano, 2020; Zheng, Allard, Hagmann, Alemán-Gómez, & Serrano, 2020).

In this study, we propose an information-theoretic approach for the analysis of synthetic and empirical brain networks with a two fold aim. The outcome of our procedure naturally accounts for the function of a system in terms of the interplay between the underlying structure and a dynamical process on the top of it, at different temporal scales, measured in bits of information required to describe the connectome state (see Materials and Methods). To this aim, we use two distinct diffusive processes for exchanging information among units: (a) a classical random walk (CRW), where the walker has no global knowledge of the connectome and performs decisions based only on local knowledge of the connectivity, while keeping a uniform probability of choosing a connection for jumping (Noh & Rieger, 2004); (b) a maximal entropy random walk (MERW), where the walker has global knowledge of the connectome and jumps through a connection, while keeping a uniform probability of choosing any trajectory on the network (Burda, Duda, Luck, & Waclaw, 2009). Thanks to these distinct dynamics, we are able to describe the network state from two distinct perspectives: one where only local knowledge is used to explore the connectome (CRW) and one where global knowledge is used instead (MERW).

On the one hand, we use our framework to compare a large set of real connectomes from healthy subjects against a selected pool of network models, characterized by distinct structural features and increasing amount of complexity. Specifically, we built 400 synthetic brain networks based on four generative models, that is, the Erdős–Rényi model (Erdős & Rényi, 1959), the configuration model (Newman, 2017), the stochastic block model (Holland, Laskey, & Leinhardt, 1983), and the hyperbolic model (Papadopoulos, Kitsak, Serrano, Boguñá, & Krioukov, 2012). These generative models (Betzel & Bassett, 2017) allow for obtaining samples of synthetic data (networks) while maintaining some specific features of empirical connectome (see Materials and Methods for a more detailed illustration of the generative models considered for this work). On the other hand, we compare the function in healthy subjects against the one in patients at different stages of dementia, namely mild cognitive impairment (MCI) and Alzheimer’s disease (AD), from a network information theory perspective.

Here we use spectral entropy, based in statistical physics and information theory—field devoted to the study of transmission, processing, extraction, and utilization of information—to investigate the structure of human brain networks. In general, the spectral entropy captures the complexity of a system in terms of the mixedness of information flow through the network. This method provides a more comprehensive analysis for the comparison of brain networks than standard techniques, since it is not limited to consider a small set of network indicators (e.g., centrality, clustering, modularity, and so forth) and their joint probability distribution, as it accounts for the contribution of the whole network state encoded into a density matrix (De Domenico & Biamonte, 2016; Ghavasieh, Nicolini, & De Domenico, 2020), a mathematical representation of the system which shares important physical and information-theoretic similarities with its counterpart widely used in quantum statistical physics (see Methods for details). Moreover, it has been systematically shown that the spectral entropy framework performs better than the traditional methods, previously introduced to investigate information dynamics within complex structures, in characterizing the global aspects of complex networks (Su, Chen, Pan, & Zeng, 2021).

We find that simple models, like the Erdős–Rényi and configuration models, have smaller spectral entropy at the mesoscale, where mid- or long-range communications between the nodes are considered, and, consequently, require a significantly smaller amount of bits (up to 1.5 bits) for their description than empirical human brains from healthy individuals. Conversely, degree-corrected stochastic block models and hyperbolic models (see Methods), accounting for the inferred modular structure of the connectome and its latent hyperbolic geometry, respectively, provide similar descriptions of the network state, with differences smaller than 1 bit. It is worth remarking that the geometry-driven model exhibits higher information entropy for increasing temporal scale—that is, moving from the mesoscale to the macroscale—denoting a larger persistence of information flow—that is, entropy tends to decay slower with Markov time—in this type of networks, at variance with stochastic block models and empirical connectomes. Results are compatible when the two types of dynamics, CRW and MERW, are considered. In general, *p* values from statistical tests indicate the mesoscale as the suitable scale to highlight differences (and similarities) between empirical data and synthetic models. Moreover, we find out that, considering the MERW dynamics, the stochastic block model can significantly reproduce the empirical brain across all scales.

When applied to connectomes obtained from healthy and unhealthy subjects, we identify significant differences between healthy individuals and the ones affected by AD at the microscale (adjusted *p* value smaller than 0.1%; maximum posterior probability smaller than 1%) and at the mesoscale (adjusted *p* value smaller than 1%; maximum posterior probability smaller than 10%), in the case of CRW. The results are confirmed, at one order of magnitude larger, for MERW and only at the microscale. Remarkably, our approach is able to capture this difference despite the fact that the topologies of the two groups exhibit a certain amount of similarity with respect to more traditional network indicators.

In the final section, we describe the interpretation of our results from a neuroscience perspective, highlighting that our approach is well suited to capture the multiscale nature of neural dynamics that is embedded in the hierarchical modular organization of brain structure. Furthermore, our method allows us to identify precisely the scale at which abnormalities can alter information flows when studying brain network in patients with AD.

## RESULTS

### Information-Theoretic Analysis of Human Brain Networks

Before discussing our results, it is important to introduce a few basic concepts that will be used in the following. Let us frame our problem in terms of a communication process, where one encodes the description of a complex network to transmit it through some noiseless communication channel to a receiver, who has to decode the corresponding information, in bits, in order to reconstruct the original network. Since the channel is assumed to be noiseless, it has maximum information capacity, that is, the mutual information between the sent and received information is maximum. Note that the communication we are referring to should not be confused with signaling or communication among distinct areas of the brain.

**Figure 1.**
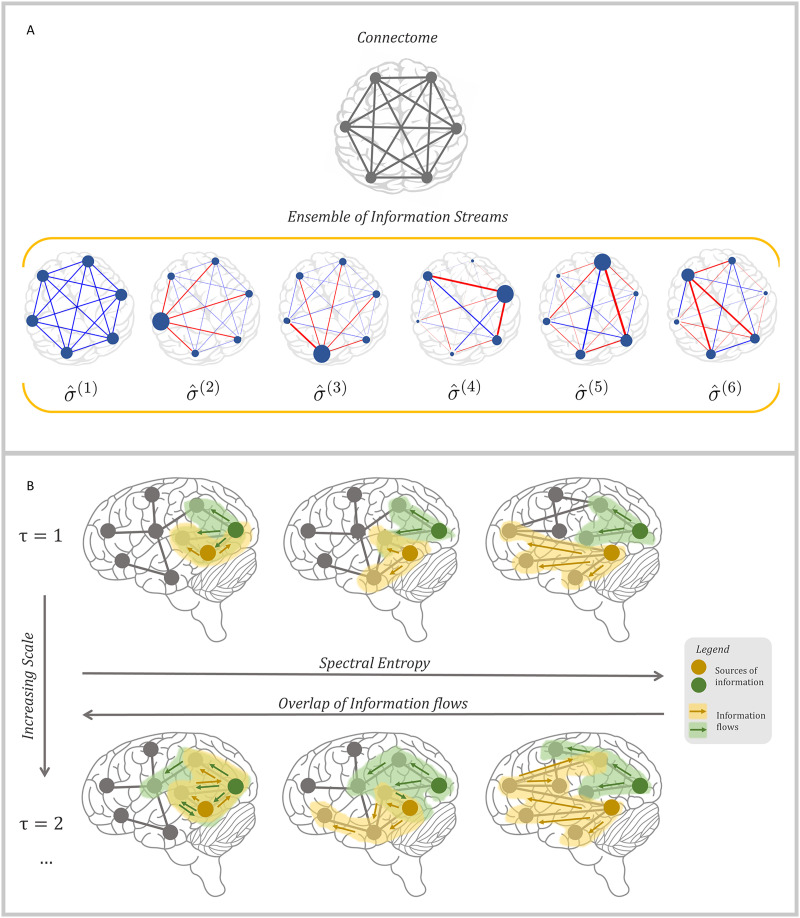
Illustrating network information entropy in the case of a human connectome. (A) A schematic view of the human connectome is presented, as a fully connected network with six nodes. Information dynamics within the connectome is regulated by an ensemble of information streams, mathematically shown as $\hat{\sigma }$^(*ℓ*)^, *ℓ* = 1, 2, … 6. The way each stream contributes to the flow of information is represented as a diagram, where blue and red arrows, respectively, represent positive and negative fluxes and the size of each node represents the amount of field trapped on top of the node. (B) Snapshots of possible functional diversity in a schematic connectome at two different Markov times (i.e., *τ* = 1 above and *τ* = 2 below). From left to right spectral entropy increases as the overlap of information flows decreases. Colored nodes encode the sources of information, shaded areas encode the flows of information, colored arrows the directions of such flows.

One way to quantify the average number of bits required to describe a network state *G* is to build the corresponding density matrix $\hat{\rho }$*_τ_*(*G*) and calculate the spectral entropy S*_τ_*(*G*), mathematically equivalent to the von Neumann entropy of an entangled quantum system (De Domenico & Biamonte, 2016). Here, the parameter *τ* indicates the Markov time of the dynamical process used to propagate information among nodes (see Materials and Methods for details): the idea is that an ensemble of signals, whose dynamics is governed by a propagator, is sent from each node to the others for a time *τ* and contributes to collect information about the underlying topology, therefore reducing uncertainty about the structure. In fact, at time *τ* = 0, no signal propagates and, consequently, the entropy is maximum because no information at all is available about network structure. Recently, it has been shown (Ghavasieh et al., 2020) that the density matrix describes the trajectories of information flow through the network and the entropy provides a measure of diversity of information dynamics in the system (see Figure 1 for an illustration). Interestingly, one can show that a network’s topological complexity, like the presence of modularity or hierarchy, can boost the diversity of information dynamics within the system and the functional diversity of nodes as senders of information (Ghavasieh et al., 2020)—that is, the modularity separates the groups of nodes from each other and the hierarchy differentiates between the groups, both making asymmetries between the nodes as senders and receivers of information and diversifying the trajectories of information flow within the system. Here, we go beyond the analysis of synthetic networks and investigate real connectomes, in comparison to null and generative models. To avoid confusion, it is worth remarking that the entropy is a macroscopic descriptor of the system as a whole, that is, it does not quantify pairwise information transfer between nodes.

Another desirable feature of this framework is that one can vary *τ* to characterize the network state at different scales, from microscopic (*τ* ∼ 1) to mesoscopic (*τ* ∼ $\sqrt{N}$) and macroscopic (*τ* ∼ *N*). Note that the scales we are referring to are topological, but the tunable parameter used to span from the microscopic to the macroscopic one is of temporal nature, since it is the time required by a dynamical process defined on the top of the network, such as a random walk, to propagate information. More specifically, we use the temporal evolution of a statistical field to explore the topological scales of the connectome, a procedure successfully adopted for the analysis of other complex systems, from the human proteome (Ghavasieh, Bontorin, Artime, Verstraete, & De Domenico, 2021), to the human microbiome (De Domenico & Biamonte, 2016) and to social and transportation systems (Ghavasieh et al., 2020).

For instance, a network of size *N* with no connectivity at all would have an entropy equal to *log*_2_*N* bits, the maximum attainable value, whereas a fully connected network (i.e., a clique), would have the lowest possible entropy, tending to 0 bits in the limit of large *τ*.

We consider two distinct dynamical processes, namely CRW and MERW (see Materials and Methods for details), and use the variation of spectral entropy with Markov time *τ* to characterize synthetic and empirical human brain networks across multiple scales. The persistence of information flow is characterized by the decay of the spectral entropy: the slower the decay, the more persistent the flow through the network.

### Probing Synthetic Models of the Human Brain

Our first analysis concerns with quantifying the differences between empirical connectomes from healthy subjects, as measured from 196 individuals within the Nathan S. Kline Institute - Rockland Sample (see Materials and Methods), and synthetic networks obtained from a pool of generative models (see Materials and Methods for details).

**Figure 2.**
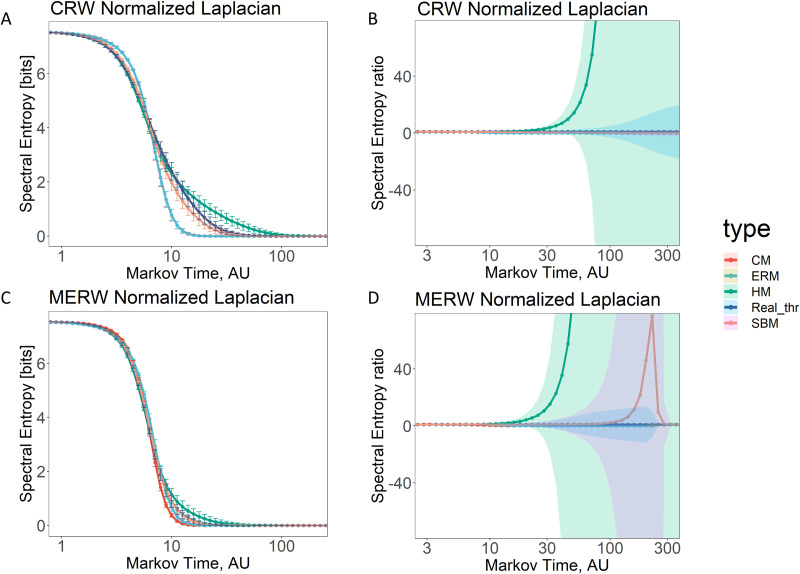
Persistence of information flow in human brain and generative models. In (A) and (C) we report the average value of spectral entropy varying the Markov time, *τ*, for the real data (blue line) and for all the considered generative models (encoded with colored lines), that is, Erdős–Rényi model (ERM), configuration model (CM), hyperbolic model (HM), and the stochastic block model (SBM), obtained from the classic random walk (CRW) and the maximal entropy random walk (MERW) dynamics, respectively. In (B) and (D) we report the ratio between the average value of spectral entropy—varying the Markov time—of each generative model and the value of spectral entropy of real data, by considering the classic random walk (CRW) and the maximal entropy random walk (MERW) dynamics, respectively. All the curves have been generated considering networks of 188 nodes, and each synthetic curve results from a sample of 19,600 realizations of the network. For all the plots, the *x*-axis is expressed in logarithmic scale. Shaded areas in (B) and (D) represent the error as one standard deviation.

Persistence of information flow is used to this aim: we calculate the average spectral entropy 〈*S_τ_*(*G_data_*)〉 over the whole set of subjects, as well as the average spectral entropy 〈*S_τ_*(*G_model_*)〉 over the ensemble of different independent realizations of a generative model, for each generative model separately, and for the two distinct dynamical processes separately. Results are shown in Figure 2. Specifically, results shown in Figure 2 have been generated by considering a sample of 196 subjects. For each subject we generate 100 different realizations of each generative model, resulting in 19,600 samples that are later used to estimate each synthetic curve shown in the figure. As expected, when considering the values of spectral entropy varying with Markov time, *τ*, the generative models exhibit distinct behavior across scales, and their ordering with respect to the value of entropy allows one rank them from the simplest to the most complex one. In fact, as it can be seen in Figure 2A and 2C the Erdős–Rényi model (ERM) and the configuration model (CM) require a smaller amount of bits for their description than empirical connectomes, followed by the more complex stochastic block model (SBM) and finally by the hyperbolic model (HM), for both CRW and MERW dynamics. Interestingly, the spectral entropy of the empirical brain lies between these last two more complex generative models, providing an indication of its possible mixed nature, interpolating between the modular feature encoded by SBM and the latent geometry encoded by HM. This results is robust across the two type of considered dynamics, CRW and MERW. It is worth noticing that differences between the spectral entropy of the connectomes and their synthetic counterpart obtained from generative models are visible while spanning from the micro- to mesoscale (*τ* ≈ $\sqrt{N}$) and that these differences are amplified at the mesoscale ($\sqrt{N}$ ≤ *τ* ≤ *N*), where *N* is the number of nodes of each network and is equal to 188 (thus, *microscale* < $\sqrt{188}$, $\sqrt{188}$ ≤ *mesoscale* < 188 and *macroscale* ≥ 188). The fact that spectral entropy values remain higher for increasing Markov time denotes a larger persistence of information flow, that is, a slower entropy decay, as in the case of the hyperbolic model. To further appreciate the differences between the entropy of synthetic and empirical networks we compute the entropic ratio *r_τ_*(*G_model_*, *G_data_*|RW) = 〈*S_τ_*(*G_model_*)〉/〈*S_τ_*(*G_data_*)〉 for each value of *τ*, generative model and random walk (RW) process (see Figure 2B and 2D).

In this case, differences are visible at the mesoscale and are amplified at the macroscale (*τ* > *N*). CRW dynamics show an important difference between the empirical brain and the hyperbolic model, highlighted by an high entropic ratio. This difference also appears when considering MERW dynamics, which, in addition, reveals the deviation of the SBM from the empirical brain at the macroscale, highlighted by a high entropic ratio.

**Figure 3.**
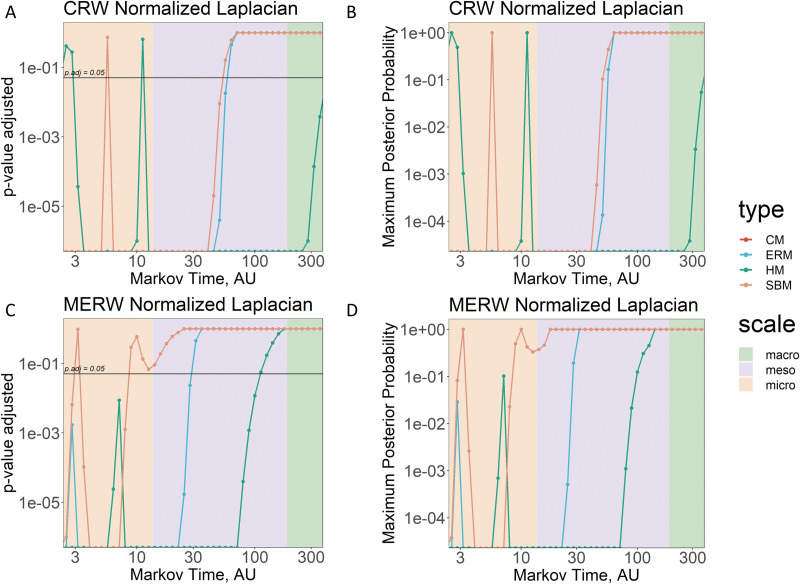
Identifying significant differences between human brain networks and generative models. In (A) and (C) we display the adjusted *p* value resulting from the *t* test between real data and all the considered generative models (encoded with colored lines), that is, Erdős–Rényi model (ERM), configuration model (CM), hyperbolic model (HM), and the stochastic block model (SBM), by considering the classic random walk (CRW) and the maximal entropy random walk (MERW) dynamics, respectively. In (B) and (D) we display the values of maximum posterior probability recalibrated from the adjusted *p* values. All plots are expressed in log-log scale. Shaded areas represent in the order the micro-, meso-, and macroscale coincident with $\sqrt{N}$ (from micro to meso) and *N* from meso to macroscale, with *N* = 188. It is to be noticed that there is an overlap between the ERM and the CM.

Leveraging on the multiresolution nature of our information-theoretic approach, we tested if there are significant differences between the empirical connectomes and their pool of generative models, by considering the values of spectral entropy at (and across) different scales defined by *τ*. Results of *t* tests between spectral entropy values coming from real data and synthetic models are provided in terms of adjusted *p* values and maximum posterior probability, and are reported in Figure 3. At the microscale, all generative models are significantly different from the human brain networks they attempt to reproduce when considering the CRW dynamics, except for a few values of spectral entropy in HM and SBM. Instead, MERW dynamics show significant similarity between real data and the stochastic block model not only at the microscale but also across the mesoscale and macroscale. For *τ* ≥ 30, above the mesoscale the synthetic networks, except for the ones generated by HM, are significantly similar to the empirical ones, when considering CRW. In the case of HM, the similarity is well established in the macroscale. In the case of MERW dynamics, the similarity with HM emerges slightly before the transition from the mesoscale to the macroscale, and across the macroscale. To further strengthen our results, we report, as well, the values of maximum posterior probability, obtained by recalibrating the *p* values adjusted (see Materials and Methods), in Figure 3B and 3D. Under some specific assumptions (see Materials and Methods), this corresponds to the error probability in rejecting the null hypothesis *H*_0_—the generative models reproduce the real data—from a Bayesian perspective.

To sum up, the mesoscale seems to be the suitable scale for distinguishing the differences (and the similarities) between the empirical brain and its possible generative models, since at the microscale the real data are significantly different from all the synthetic models, while at the macroscale there are not significant differences between data and models. Curiously, when considering the MERW dynamics, the empirical brain can resemble a stochastic block model across all scales, thus supporting the broad application of community detection algorithms and stressing their importance for the analysis and the understanding of the human brain.

### Information Capacity at Different Stages of Dementia

Here, we wonder if we can use the same framework to identify differences between healthy subjects and patients at different stages of dementia, namely mild cognitive impairment (MCI) and Alzheimer’s disease (AD). For details about the dataset used for this analysis, we refer to Materials and Methods.

**Figure 4.**
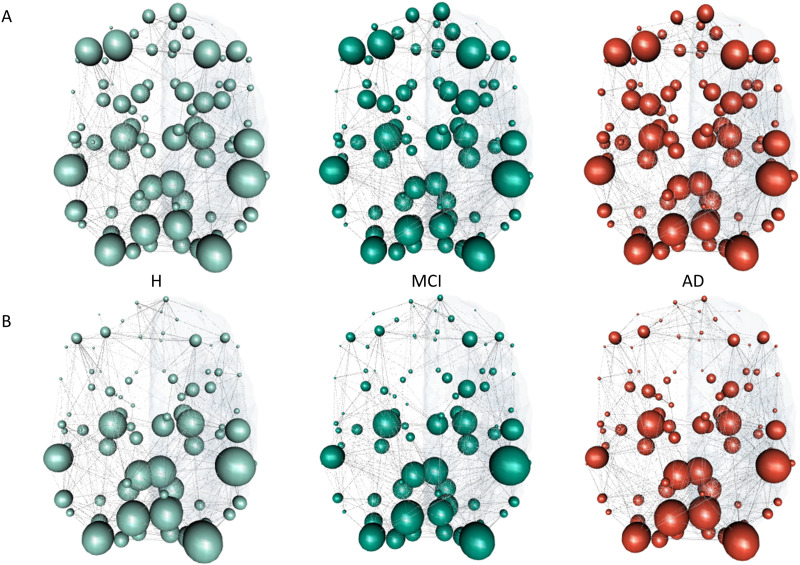
Brain maps of the steady-state distribution for the CRW and MERW dynamics in healthy brain (H) and at different stages of dementia (MCI and AD). In (A) we report the steady state for the CRW dynamics, while in (B) the steady state for the MERW dynamics. The size of the node encodes the value of the steady state corresponding to the leading eigenvector of the process.

To spatially characterize different diffusion processes (i.e., CRW and MERW) on top of the network in healthy brain (H) and at different stages of dementia (MCI and AD), we provide brain maps encoding the steady state of the two considered dynamics, corresponding to the leading eigenvector of the transition matrix defining the process (see Figure 4).

**Figure 5.**
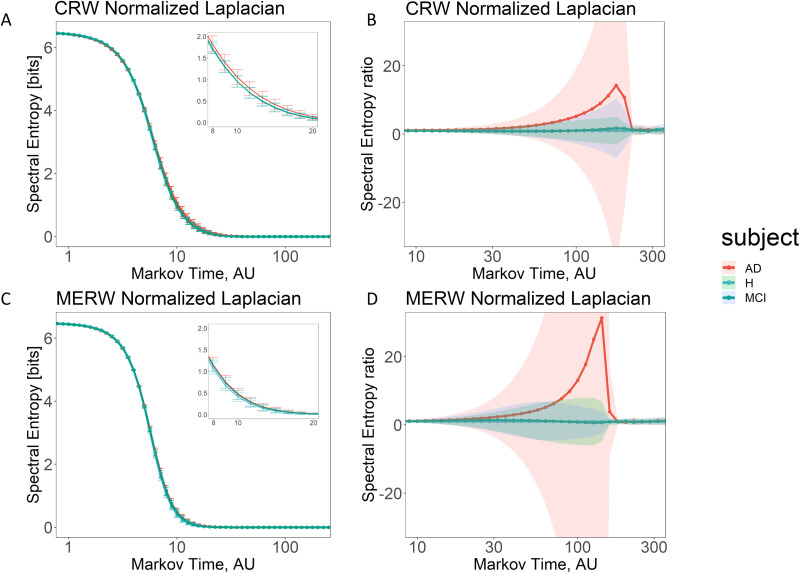
Persistence of information flow in healthy brain (H) and different stages of dementia (MCI and AD). (A and C) We report the average value of spectral entropy varying the Markov time, *τ*, for healthy subjects (H, light-blue line) and different stages of dementia (MCI in teal and Alzheimer’s disease in red) by considering the classic random walk (CRW) and the maximal entropy random walk (MERW) dynamics, respectively. The inset on the top right represents a zoom on the region at *τ* = [8 − 20]. In (B) and (D) we report the ratio between the average value of spectral entropy—varying the Markov time—of each stage of dementia and the value of spectral entropy of healthy subjects, by considering CRW and MERW dynamics, respectively. For all the plots, the *x*-axis is expressed in logarithmic scale. Shaded areas (B and D) represent the error as one standard deviation.

Using the same approach as before, we show the results in Figure 5. At the turn of micro and mesoscale, when considering CRW dynamics, the values of spectral entropy in the connectome of Alzheimer’s disease patients show some differences from both healthy subjects and MCI patients. Intriguingly, the connectome of AD patients exhibits a (slightly) higher spectral entropy than the ones of healthy and MCI subjects, denoting a larger persistence of information flow. As in the previous case, to further highlight differences between healthy and different stages of dementia, we compute the ratio between the corresponding values of spectral entropy (see plots of Figure 5B and 5D). Differences between AD patients and the other two considered groups of subjects (healthy and MCI) are visible at the mesoscale and are amplified at the macroscale, while no differences appear between MCI and healthy. These results are in agreement for the two dynamics, CRW and MERW.

**Figure 6.**
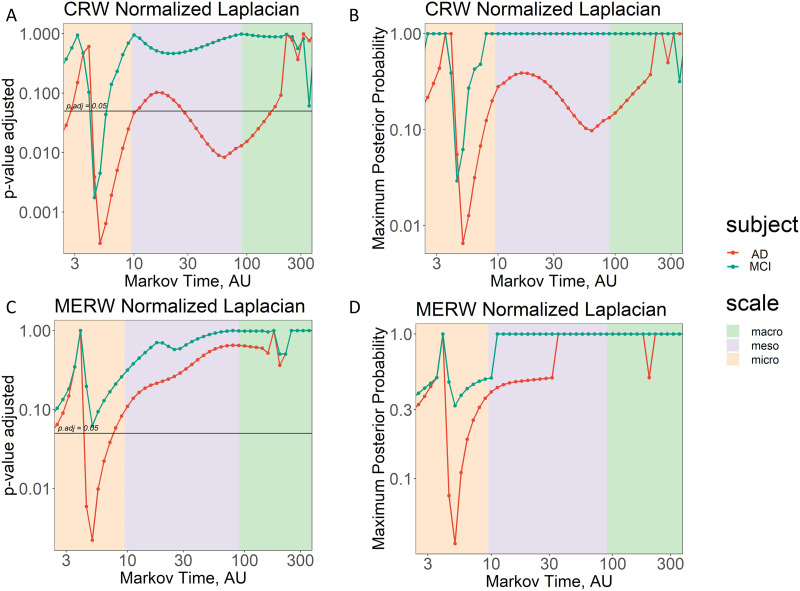
Identifying significant differences between human brain networks in health and disease. (A and C) We display the adjusted *p* value resulting from the *t* test between different stage of dementia (MCI in teal and Alzheimer’s disease in red) and healthy subjects for each value of spectral entropy varying with Markov time, *τ*, by considering the classic random walk (CRW) and the maximal entropy random walk (MERW) dynamics, respectively. Horizontal lines mark the *p* value adjusted at 0.05. (B and D) We display the values of maximum posterior probability recalibrated from the adjusted *p* values. All the plots are expressed in log-log scale. Shaded areas represent in the order the micro-, meso-, and macroscale coincident with $\sqrt{N}$ (from micro to meso) and *N* from meso- to macroscale, with *N* = 90.

Also in this case, we tested the significance of the differences between the spectral entropy of the two stages of dementia and healthy controls by means of *t* tests; results are displayed in Figure 6. According to the adjusted *p* values obtained when considering the CRW dynamics (see plot of Figure 6A), the spectral entropy in AD patients is significantly different from the one of healthy controls for most of the Markov time values at the micro-, meso-, and macroscale. For what concerns the MERW dynamics (see plot of Figure 6C), the same is true only at the microscale. Interestingly, the only significant differences between values of spectral entropy in MCI and in healthy controls arises at the microscale and only for the CRW dynamics. Finally, resulting values of maximum posterior probability strengthen the results at the microscale for the AD patients (probability of the error < 1%) when considering CRW dynamics.

## DISCUSSION

We presented a multiresolution analysis of the human brain building on statistical physics and information theory of complex networks.

Patterns of distributed activity, or modes, of the brain are neural dynamics unfolding on anatomical connectivity structures (Honey, Kötter, Breakspear, & Sporns, 2007). The topological structure of the brain has been investigated using many functional and structural neuroimaging datasets (Bullmore & Sporns, 2009; K. Friston, Kahan, Razi, Stephan, & Sporns, 2014; K. J. Friston, 2009; Sporns, Chialvo, Kaiser, & Hilgetag, 2004) that have established that brain regions have a modular functional organization and are also connected in a way that permits the emergence of whole-brain processes like attention, cognition, and behavior. In other words, functionally distinct brain areas have a hierarchical organization that permits integration at different topological scales (Bassett et al., 2008; Meunier et al., 2009). A typical example of a task that is implemented at different scales is invariant visual object recognition, which relies on a hierarchically organized set of visual cortical areas whose competition, biased by attention, is implemented locally but gradually increases thanks to the hierarchical nature of the network (Deco & Rolls, 2004, 2005). In this context, to simultaneously capture the state of the network at different scales is crucial to fully understand a neural process. In this paper we focused on resting-state structural data but further research, investigating behavioral or cognitive functional tasks, can benefit from our approach. In particular, we could investigate how the functional role of brain regions changes in resting state with respect to specific behavioral or cognitive tasks, or how functional alterations are displayed at multiple brain scales in nonhealthy subjects.

We exploited the information flow among system units restricted by the underlying connections to gain insights into the different functional role of brain regions at multiple scales. Here we used classical and maximal entropy random walk processes to explore the topological scale of the networks, but other types of dynamical processes (such as synchronization processes) on the top of the system may be considered in further works, since the framework is very flexible. We first use our method to compare network models that have been widely used in the literature to describe brain organization and we found that at the microscale, all the tested models are not suitable to describe real data. We hypothesize that, although MRI resolution preclude analysis about functional specialization within the dendritic tree or cortical macrocolumn (K. J. Friston, 2009), their layered structures could strongly affect the results at low spatial scale. On the contrary, the mesoscale is the most suitable resolution to compare network topologies. We found that real connectomes have features of the stochastic block model and the hyperbolic model, where the former is representing the aforementioned brain modular organization of brain areas and the latter takes into account latent geometry in the communication flows among them.

Several previous studies have explored the mechanisms that control communication dynamics in brain networks (Amico et al., 2021; Avena-Koenigsberger, Misic, & Sporns, 2018; Hahn, Ponce-Alvarez, Deco, Aertsen, & Kumar, 2019). Some models suggest that neural units have a knowledge of the whole network topology and convey information from a source to a predetermined target using (multiple) shortest paths (Avena-Koenigsberger et al., 2017; Goni et al., 2014). Other models, instead, do not make assumptions about global knowledge of network topology and propose that communication flows are ruled only by local knowledge of the distance between cortical regions (Seguin, van den Heuvel, & Zalesky, 2018). In our work we explore both frameworks using two different diffusive processes to describe information flows among units: in the classical random walk we assume that communication dynamics only require local knowledge of the connectivity, whereas in the max-entropy random walk process we hypothesize that the walker uses global knowledge of the connectome to explore it. We found that the two approaches are compatible and that spectral entropy values are similar when we consider the two types of dynamics on top of stochastic block model and hyperbolic model. Those results are in agreement with the aforementioned study that shows how specific brain topological and geometrical properties lead to comparable efficiency in network communication with or without centralized knowledge.

Finally, our approach was used to investigated alterations in brain network topology in patients with AD. Here we can avoid any assumption regarding the network generative model that better describes real data and we can focus on changes in information flows that characterize AD brain networks at different scales. Previous studies used standard network analysis indicators to report abnormalities in the connectivity between different brain areas, and specifically found an increased connectivity at spatial scales lower than brain lobe and postulated a mechanism of compensation associated with cognitive impairment (Penny, Iglesias-Fuster, Quiroz, Lopera, & Bobes, 2018; Reuter-Lorenz & Cappell, 2008; Sheng et al., 2021; Yao et al., 2010). Our results are in agreement with the aforementioned studies, since they show a significant increase in persistence of information flows at microscopic scales in AD patients with respect to healthy subjects. Furthermore, by taking simultaneously into account the whole network state, they suggest that compensation mechanisms may act at smaller topological scales than previously hypothesized.

We conclude by outlining some limitations of our approach. Statistical physics of complex information dynamics has been shown to be a powerful framework to attack a range of problems in the domain of complex systems. Yet, it is worth mentioning that the computational cost for calculating spectral entropy is still relatively high, being of the same order of the complexity of an eigenvalue problem. Therefore, our method would perform slower than more standard techniques in the case of very large networks, that is, for sizes above 10,000 nodes; for smaller networks, the method is fast enough. The robustness of our analysis should be assessed also by analyzing other empirical datasets to further clarify how our entropy measures can be affected by the definition of distinct brain areas and can evolve when considering nonstationary (no resting state) brain activities. Finally, although the results presented in this work are promising for the investigation of dementia, the clinical usability of our approach requires further investigation.

## MATERIALS AND METHODS

### Data

In this work we rely on two structural connectivity data sets:

1.  A structural connectivity data set provided by the Nathan S. Kline Institute - Rockland Sample (NKI-RS), consisting of 196 healthy subjects (USC Multimodal Connectivity Database: https://umcd.humanconnectomeproject.org/).
2.  A structural connectivity data set of 71 subjects from Lin et al. (2019), among which 22 with Alzheimer’s disease, 23 affected by mild cognitive impairment, and 26 healthy controls.

The first data set (Nooner et al., 2012) consists of resting-state structural data, that represent physical structure of brain networks, of 196 healthy subjects at rest without any mental or physical disorder. The sample is made up of 114 males and 82 females and the age range is rather large: the youngest person is 4 and the eldest is 85. By looking at the age variable distribution, the first quartile is equal to 20, and the third quartile is equal to 47; for this reason, the sample can be considered representative of age variability. The NKI-RS has been designed as a community-ascertained sample and the representativeness is maximized according to demographic characteristics of the United States. Brain structural networks are mapped with diffusion tensor imaging measures (137-direction, 2 mm isotropic), provided by the Center for Magnetic Resonance Research at the University of Minnesota for the Human Connectomes Project. All data were publicly shared through the Collaborative Informatics and Neuroimaging Suite (COINS) developed by the Mind Research Network.

The second data set consists of structural networks reconstructed from diffusion tensor imaging data. In particular, to reconstruct the networks, Lin et al. applied a streamline-based fiber tracking algorithm on voxelwise diffusion tensors with these set parameters: random whole-brain seeding, 200,000 reconstructed streamlines, anisotropy threshold of 0.15, angular threshold of 45°, and streamline length between 30 and 300 mm (for more details about this data set please refer to Lin et al., 2019). Here, the nodes of the brain networks correspond to the 90 cerebral regions from the automatic anatomical labeling (AAL) template (Tzourio-Mazoyer et al., 2002), while the edges are quantified by computing the fractional anisotropy (FA) along the interconnected streamlines between two different AAL regions. According to Lin et al. (2019), measurements obtained from tract-specific metrics (e.g., fractional anisotropy and diffusivity) reveal themselves to be more sensitive and interpretable than those obtained from metrics based on streamline count. These findings motivate our choice to rely on data obtained from a fractional anisotropy tract-specific metric for building the empirical brain networks.

Fractional anisotropy ranges from 0 to 1, where 0 means that diffusion is isotropic and 1 that diffusion occurs along one axis. To establish the presence of links in a binary way, and to avoid, at the same time, using arbitrary thresholds, the links of the networks used in this work are the result of a sampling assuming that the probability of existence of each link is uniformly distributed. Specifically, we defined the link between *i* and *j* in the FA dataset as *w_ij_* and, interpreting *w_ij_* as a probability, we extract a random number *r* from a uniform distribution *U*(0, 1) and we assign the binary link according to the Heaviside Θ as *a_ij_* = Θ(*w_ij_* − *r*). In other words, when the value of FA is greater than the corresponding random value the link exists, otherwise it is discarded. In this case, the sampling is well suited given the FA values bounded between 0 and 1 and can be safely interpreted as the probability of a link to exist. In fact, since FA values measure the degree of anisotropy of diffusion occurring on a given tract and being such values bounded between 0 and 1—where 0 means isotropic diffusion and 1 anisotropic diffusion—they can be safely interpreted as the probability to have a structural connections among brain regions. The choice of avoiding to adopt a specific threshold is motivated by recent studies showing that, in the case of probabilistic or correlation networks, it is desirable to account for the intrinsic uncertainty in the existence of each link (Raimondo & Domenico, 2021).

### Generative Models

Generative models are statistical processes allowing one to obtain a sample of synthetic data. The synthetic networks obtained through such processes can share some properties with the observed data, and this procedure is guaranteed by the use of parameters that are usually obtained by fitting the observed data. In this study, we consider four different types of generative models; for each type, we fit the underlying parameters of each model for each empirical connectome separately, and generate 100 independent realizations, to obtain an ensemble of synthetic networks for each connectome. Therefore, we have a total of 400 synthetic networks for each empirical network.

The Erdős–Rényi model (ERM) generates random graphs with the same number of vertices and the same number of edges of the real network. These topological features are preserved each time the model is produced, whereas the network structure randomly changes.

The configuration model (CM) reproduces the degree distribution of the real network, preserving the the degree of the nodes while avoiding multiple edges; in the literature, this model is also known as degree-preserving random rewiring model (Maslov & Sneppen, 2002). Parameters used to fit this data-driven model are as many as the number of nodes *N*, and each represents the corresponding node degree *k_i_* (*i* = 1, 2, …, *N*). From each node, *k_i_ stubs* (edge halves) start and, by changing the connectivity pattern, link to different nodes, obtaining a network with no topological correlations which preserve the observed connectivity.

The stochastic block model (SBM) allows one to define an ensemble of random models that reproduce the mesoscale organization present in the real network. A block consists of a group of nodes that have a higher likelihood of being connected among them than making external connections with nodes from other groups. Here, we use *graph-tool* (Peixoto, 2019), an efficient Python module for statistical analysis of graphs and network manipulation, to fit the degree-corrected SBM.

The hyperbolic model (HM) is based on two important parameters, the popularity and the similarity (Papadopoulos et al., 2012), whose trade-off is responsible for the network structure: the two parameters are physically formalized and geometrically interpreted, and their product is optimized in order to obtain connections in the network. To fit this model, we use the *Mercator* method (García-Pérez, Allard, Serrano, & Boguñá, 2019), which maps real complex networks into a hyperbolic geometric space, which is able to provide a more accurate interpretation of the connectome structure than Euclidean geometry. We use Mercator (Network Geometry - Mercator, 2020) to generate this class of synthetic networks (Allard & Serrano, 2020).

### Random Walks on Connectomes

Information flow in complex networks, such as human connectomes, has been modeled by diffusive processes like random walks (Masuda, Porter, & Lambiotte, 2017). As Markovian processes, different types of random walks are defined in terms of transition matrices encoding the probability of jumps from nodes to neighbors. In this work, we use two important types, including the classical random walk (CRW) (Noh & Rieger, 2004) and maximal entropy random walk (MERW) (Burda et al., 2009).

For both types of dynamics, the Laplacian matrix is defined by L = **I** − **T**, where **T** is the transition matrix governing random walk dynamics and **I** is the identity matrix. Let us assume that the *i*-th components of the vector **p**(*τ*) indicate the probability to find the random walker in node *i* at time *τ*. The evolution of the probability vector is governed by the master equation

$$p\left(\tau +1\right)=p\left(\tau \right)T.$$ (1)

In the continuous-time approximation, Equation 1 reduces to:

$$\frac{\partial p\left(\tau \right)}{\partial \tau }+p\left(\tau \right)L=0,$$ (2)

with solution given by **p**(*τ*) = **p**(0)*e*^−*τ*L^.

In a classical random walk on a binary network, the transition matrix is defined as $T_{ij}^{\left(CRW\right)}$ = *A_ij_*/*k_i_*, where *k_i_* is the degree of *i*-th node and ***A*** is the adjacency matrix. In MERW the transition matrix is defined in terms of the largest eigenvector of the adjacency matrix. Assume the eigenvalues of the adjacency matrix are ordered as *a_ℓ_*, *ℓ* = 1, 2, …, *N*, where *a_N_* has the maximum value, and their corresponding eigenvectors are given by **q**^(*ℓ*)^. The transition matrix, in this case, is given by $T_{ij}^{\left(\text{MERW}\right)}$ = $\frac{A_{ij}}{a_{N}}\frac{q_{j}^{\left(N\right)}}{q_{i}^{\left(N\right)}}$. One of the interesting features of MERW is that the probability of a transition from one node to another within *τ* steps of time is independent of the intermediate transitions and all trajectories from *i*-th to *j*-th node with the length of *τ* are equiprobable.

The Laplacian matrix for CRW is L^(*CRW*)^ = **I** − **T**^(*CRW*)^, while for MERW it is defined by L^(*MERW*)^ = **I** − **T**^(*MERW*)^. Therefore, each dynamical process can be obtained from Equation 2 by choosing the relevant Laplacian matrix.

### Statistical Physics of Information Dynamics

Characterizing the flow of information between nodes in a complex network is challenging, requiring a deep understanding of the network topology, relevant dynamical processes, and the interplay between them. Recently, a statistical field theory has been introduced to describe the information flow between the components of complex systems in terms of the dynamics of a field on top of the network, moving among nodes. In this framework, for a network denoted as *G*, the dynamical process governing the flow can be described in terms of a general differential equation, which, after linearization, reduces to a Schrodinger-like equation with a quasi-Hamiltonian $\hat{H}$(*G*) and the propagator *e*^−*τ*^$\hat{H}$^(*G*)^ that determines the flow trajectories at time *τ*. Furthermore, the propagator can be eigen-decomposed to obtain an ensemble of operators acting like information streams, directing the flow of the field from unit to unit (Ghavasieh et al., 2020). The topological factors, the type of the quasi-Hamiltonian and *τ* affect the size of the streams and, consequently, each stream can be active or nonactive (i.e., having negligible size) under a specific system configuration.

To study the macroscopic properties of these microscopic interactions between the nodes, it has been shown that a superposition of the information streams, weighted by their activation probabilities, provides a Gibbsian-like density matrix describing the state of the system:

$${\hat{\rho }}_{\tau }\left(G\right)=\frac{e^{-\tau \hat{H}\left(G\right)}}{Z_{\tau }\left(G\right)},$$ (3)

where *Z_τ_*(*G*) = *Tr*[*e*^−*τ*^$\hat{H}$^(*G*)^] plays the role of the partition function and is related to the transport properties of the network (Ghavasieh & Domenico, 2020). Using the above density matrix, one can quantify the mixed-ness of the information streams in terms of the von Neumann entropy as

$$S_{\tau }\left(G\right)=-Tr\left[{\hat{\rho }}_{\tau }\left(G\right)\log_{2}{\hat{\rho }}_{\tau }\left(G\right)\right],$$ (4)

which is also a measure of diversity of the flow dynamics. The maximum value for the von Neumann entropy of the system is log_2_ *N* corresponding to the state where all information streams are active with the same size and to capture the dynamics, one needs to consider all the streams. At large temporal scales *τ*, the entropy of a connected network is expected to decay, as the distribution of the field becomes less dependent on the initial conditions. Interestingly, it has been shown that the von Neumann entropy can be used to measure the functional diversity of nodes as senders of information (Ghavasieh et al., 2020). In fact, in a system where the overlap between the flow distribution originated from different nodes is high, we get lower values for the entropy.

It is worth noting that if the dynamical process is continuous diffusion governed by the combinatorial Laplacian, the von Neumann entropy obtained from the above statistical field theory is equal to the spectral entropy (De Domenico & Biamonte, 2016), introduced to analyze complex networks from an information-theoretic perspective.

Here, we consider random walk dynamics as a proxy for information transport in human connectomes. Therefore, the quasi-Hamiltonian equals L^(*CRW*)^ for classical random walk and L^(*MERW*)^ for maximal entropy random walk.

### Maximum Posterior Probability

To quantify the statistical significance of the differences (or similarities) within the connectomes coming from two distinct groups (e.g., empirical data vs. model, control vs. disease, etc.) considered for this work, we performed pairwise *t* tests, adequately corrected for multiple test comparison. In particular, we tested two distinct null hypotheses (*H*_0_): (a) the generative models reproduce the real data (dataset 1) and (b) the spectral entropies in healthy and nonhealthy brains are equal (dataset 2). Since we are performing multiple tests, that is, we tested real data against the four generative models and the healthy controls against different stages of dementia, we adjusted the resulting *p* values by means of the Bonferroni–Holm method. All pairwise *t* tests are performed by considering a 95% confidence interval.

To avoid confusion in the interpretation of the *p* values, here we use a Bayesian approach for *p* value calibration proposed by Sellke, Bayarri, and Berger (2001), so that *p* values can be interpreted from both a frequentist and a Bayesian perspective. Specifically, we compute the Bayes factor as:

$$B\left(p\right)=-ep\log\left(p\right)$$ (5)

for *p* < 1/*e*, which corresponds to the lower bound on the odds provided by the data for *H*_0_ and *H*_1_, the latter being the alternative hypothesis. If we consider the frequentist error probability of rejecting *H*_0_ when it is true (type I error), the calibration is given by

$$\alpha \left(p\right)={\left(1+B^{-1}\left(p\right)\right)}^{-1}$$ (6)

where, in this case, *p* is the adjusted *p* value. Therefore, we have two possible interpretation for the outcome of this calibration. From a frequentist perspective, it precisely coincides with the error probability of rejecting a true null hypothesis. From a Bayesian perspective, it is the (maximum) posterior probability of *H*_0_ provided that the Bayes factor corresponds to the one expressed in Equation 5 and assuming that *H*_0_ and *H*_1_ have equal prior probabilities of 1/2. The results of *t* test thus can be either interpreted as the probability of rejecting the null hypothesis when it is true and as the probability of the null hypothesis itself. In other words, lower recalibrated *p* values are indicative of lower accordance between the samples that we are testing—lower maximum posterior probability—while higher recalibrated *p* values can be interpreted as higher probability of accordance between the samples under consideration.

## ACKNOWLEDGMENTS

The authors acknowledge Dr. Shih-Yen Lin, Dr. Li-Wei Kuo and the MR NeuroImaging Lab (National Health Research Institutes, Taiwan) for kindly providing access to some of the empirical datasets used in this study.

## AUTHOR CONTRIBUTIONS

Barbara Benigni: Conceptualization; Data curation; Formal analysis; Investigation; Resources; Software; Visualization; Writing – original draft; Writing – review & editing. Arsham Ghavasieh: Investigation; Methodology; Visualization; Writing – original draft; Writing – review & editing. Alessandra Corso: Data curation; Resources; Writing – original draft. Valeria d’Andrea: Investigation; Writing – original draft; Writing – review & editing. Manlio De Domenico: Conceptualization; Data curation; Investigation; Methodology; Project administration; Resources; Supervision; Validation; Writing – original draft; Writing – review & editing.

## TECHNICAL TERMS

Spectral entropy: The mixedness of information streams determined by the Von Neumann entropy of the density matrix defining the information content of the network.
Information flow: Communication of brain areas happen through the propagation of (electrochemical) signals. Diffusion processes are used as a proxy to determine the flow of information in the connectome.
Information capacity: Amount of information allowed to pass through a communication channel in a given time period.
Density matrix: A matrix encoding the state of the system obtained from the superposition of information streams weighted by their activation probabilities.
Structural connectivity: Topological interconnection of brain regions as identified through diffusion tensor imaging (DTI) techniques and summarized in adjacency matrices according to some specific indicator (e.g., fractional anisotropy).
Fractional anisotropy: Degree of anisotropy in a diffusion process bounded between 0 and 1, where 0 means isotropic diffusion and 1 represents a diffusion occurring only along one axis.
Information streams: Operators (i.e., matrices) obtained from eigen-decomposition of propagator, responsible for directing information flow through the system.
Partition function: The summation of the stream sizes, encoding each stream contribution to the overall flow, that is used to normalize the density matrix.
*P* value calibration: Recomputing the *p* value so that it can be interpreted as the probability of *H*_0_, under some specific assumptions.

## References

[bib1] Alexander-Bloch, A., Lambiotte, R., Roberts, B., Giedd, J., Gogtay, N., & Bullmore, E. (2012). The discovery of population differences in network community structure: New methods and applications to brain functional networks in schizophrenia. NeuroImage, 59(4), 3889–3900. https://doi.org/10.1016/j.neuroimage.2011.11.035, 22119652 22119652 10.1016/j.neuroimage.2011.11.035 PMC3478383

[bib2] Alexander-Bloch, A. F., Vértes, P. E., Stidd, R., Lalonde, F., Clasen, L., Rapoport, J., … Gogtay, N. (2013). The anatomical distance of functional connections predicts brain network topology in health and schizophrenia. Cerebral Cortex, 23(1), 127–138. https://doi.org/10.1093/cercor/bhr388, 22275481 22275481 10.1093/cercor/bhr388 PMC3513955

[bib3] Allard, A., & Serrano, M. Á. (2020). Navigable maps of structural brain networks across species. PLoS Computational Biology, 16(2), e1007584. https://doi.org/10.1371/journal.pcbi.1007584, 32012151 32012151 10.1371/journal.pcbi.1007584 PMC7018228

[bib4] Amico, E., Abbas, K., Duong-Tran, D. A., Tipnis, U., Rajapandian, M., Chumin, E., … Goñi, J. (2021). Toward an information theoretical description of communication in brain networks. Network Neuroscience, 1–20. 10.1162/netn_a_00185 34746621 PMC8567835

[bib5] Amico, E., & Goñi, J. (2018). Mapping hybrid functional-structural connectivity traits in the human connectome. Network Neuroscience, 2(3), 306–322. https://doi.org/10.1162/netn_a_00049, 30259007 30259007 10.1162/netn_a_00049 PMC6145853

[bib6] Assaf, Y., & Pasternak, O. (2007). Diffusion tensor imaging (DTI)-based white matter mapping in brain research: A review. Journal of Molecular Neuroscience, 34(1), 51–61. https://doi.org/10.1007/s12031-007-0029-0, 18157658 10.1007/s12031-007-0029-0 18157658

[bib7] Avena-Koenigsberger, A., Mišic´, B., Hawkins, R. X., Griffa, A., Hagmann, P., Goñi, J., & Sporns, O. (2017). Path ensembles and a tradeoff between communication efficiency and resilience in the human connectome. Brain Structure and Function, 222(1), 603–618. https://doi.org/10.1007/s00429-016-1238-5, 27334341 27334341 10.1007/s00429-016-1238-5

[bib8] Avena-Koenigsberger, A., Misic, B., & Sporns, O. (2018). Communication dynamics in complex brain networks. Nature Reviews Neuroscience, 19(1), 17–33. https://doi.org/10.1038/nrn.2017.149, 29238085 10.1038/nrn.2017.149 29238085

[bib9] Bassett, D. S., Bullmore, E., Verchinski, B. A., Mattay, V. S., Weinberger, D. R., & Meyer-Lindenberg, A. (2008). Hierarchical organization of human cortical networks in health and Schizophrenia. Journal of Neuroscience, 28(37), 9239–9248. https://doi.org/10.1523/JNEUROSCI.1929-08.2008, 18784304 18784304 10.1523/JNEUROSCI.1929-08.2008 PMC2878961

[bib10] Bassett, D. S., & Bullmore, E. T. (2016). Small-world brain networks revisited. The Neuroscientist, 23(5), 499–516. https://doi.org/10.1177/1073858416667720, 27655008 27655008 10.1177/1073858416667720 PMC5603984

[bib11] Bassett, D. S., & Sporns, O. (2017). Network neuroscience. Nature Neuroscience, 20(3), 353–364. https://doi.org/10.1038/nn.4502, 28230844 28230844 10.1038/nn.4502 PMC5485642

[bib12] Bassett, D. S., Wymbs, N. F., Porter, M. A., Mucha, P. J., Carlson, J. M., & Grafton, S. T. (2011). Dynamic reconfiguration of human brain networks during learning. Proceedings of the National Academy of Sciences, 108(18), 7641–7646. https://doi.org/10.1073/pnas.1018985108, 21502525 10.1073/pnas.1018985108 PMC3088578 21502525

[bib13] Battiston, F., Guillon, J., Chavez, M., Latora, V., & de Vico Fallani, F. (2018). Multiplex core–periphery organization of the human connectome. Journal of the Royal Society Interface, 15(146), 20180514. https://doi.org/10.1098/rsif.2018.0514, 30209045 10.1098/rsif.2018.0514 PMC6170773 30209045

[bib14] Bertolero, M. A., Yeo, B. T. T., Bassett, D. S., & D’Esposito, M. (2018). A mechanistic model of connector hubs, modularity and cognition. Nature Human Behaviour, 2(10), 765–777. https://doi.org/10.1038/s41562-018-0420-6, 30631825 10.1038/s41562-018-0420-6 PMC6322416 30631825

[bib15] Betzel, R. F. (2020). Community detection in network neuroscience. arXiv preprint arXiv:2011.06723.

[bib16] Betzel, R. F., Avena-Koenigsberger, A., Goñi, J., He, Y., De Reus, M. A., Griffa, A., … Sporns, O. (2016). Generative models of the human connectome. NeuroImage, 124, 1054–1064. https://doi.org/10.1016/j.neuroimage.2015.09.041, 26427642 26427642 10.1016/j.neuroimage.2015.09.041 PMC4655950

[bib17] Betzel, R. F., & Bassett, D. S. (2017). Generative models for network neuroscience: Prospects and promise. Journal of the Royal Society Interface, 14. https://doi.org/10.1098/rsif.2017.0623, 29187640 10.1098/rsif.2017.0623 PMC5721166 29187640

[bib18] Betzel, R. F., & Bassett, D. S. (2018). Specificity and robustness of long-distance connections in weighted, interareal connectomes. Proceedings of the National Academy of Sciences, 115(21), E4880–E4889. https://doi.org/10.1073/pnas.1720186115, 29739890 10.1073/pnas.1720186115 PMC6003515 29739890

[bib19] Buldú, J. M., & Porter, M. A. (2018). Frequency-based brain networks: From a multiplex framework to a full multilayer description. Network Neuroscience, 2(4), 418–441. https://doi.org/10.1162/netn_a_00033, 30294706 30294706 10.1162/netn_a_00033 PMC6147638

[bib20] Bullmore, E., & Sporns, O. (2009). Complex brain networks: Graph theoretical analysis of structural and functional systems. Nature Reviews Neuroscience, 10(3), 186–198. https://doi.org/10.1038/nrn2575, 19190637 19190637 10.1038/nrn2575

[bib21] Bullmore, E., & Sporns, O. (2012). The economy of brain network organization. Nature Reviews Neuroscience, 13(5), 336–349. https://doi.org/10.1038/nrn3214, 22498897 22498897 10.1038/nrn3214

[bib22] Burda, Z., Duda, J., Luck, J.-M., & Waclaw, B. (2009). Localization of the maximal entropy random walk. Physical Review Letters, 102(16), 160602. https://doi.org/10.1103/PhysRevLett.102.160602, 19518691 19518691 10.1103/PhysRevLett.102.160602

[bib23] Castellanos, F. X., & Proal, E. (2012). Large-scale brain systems in ADHD: Beyond the prefrontal–striatal model. Trends in Cognitive Sciences, 16(1), 17–26. https://doi.org/10.1016/j.tics.2011.11.007, 22169776 22169776 10.1016/j.tics.2011.11.007 PMC3272832

[bib24] Deco, G., Jirsa, V. K., & McIntosh, A. R. (2010). Emerging concepts for the dynamical organization of resting-state activity in the brain. Nature Reviews Neuroscience, 12(1), 43–56. https://doi.org/10.1038/nrn2961, 21170073 10.1038/nrn2961 21170073

[bib25] Deco, G., & Rolls, E. T. (2004). A Neurodynamical cortical model of visual attention and invariant object recognition. Vision Research, 44(6), 621–642. https://doi.org/10.1016/j.visres.2003.09.037, 14693189 14693189 10.1016/j.visres.2003.09.037

[bib26] Deco, G., & Rolls, E. T. (2005). Attention, short-term memory, and action selection: A unifying theory. Progress in Neurobiology, 76(4), 236–256. https://doi.org/10.1016/j.pneurobio.2005.08.004, 16257103 16257103 10.1016/j.pneurobio.2005.08.004

[bib27] Deco, G., Tononi, G., Boly, M., & Kringelbach, M. L. (2015). Rethinking segregation and integration: Contributions of whole-brain modelling. Nature Reviews Neuroscience, 16(7), 430–439. https://doi.org/10.1038/nrn3963, 26081790 26081790 10.1038/nrn3963

[bib28] De Domenico, M. (2017). Multilayer modeling and analysis of human brain networks. Giga Science, 6(5), gix004. https://doi.org/10.1093/gigascience/gix004, 28327916 10.1093/gigascience/gix004 PMC5437946 28327916

[bib29] De Domenico, M., & Biamonte, J. (2016). Spectral entropies as information-theoretic tools for complex network comparison. Physical Review X, 6(4), 041062. 10.1103/PhysRevX.6.041062

[bib30] De Domenico, M., Sasai, S., & Arenas, A. (2016). Mapping multiplex hubs in human functional brain networks. Frontiers in Neuroscience, 10, 326. https://doi.org/10.3389/fnins.2016.00326, 27471443 27471443 10.3389/fnins.2016.00326 PMC4945645

[bib31] De Domenico, M., Solé-Ribalta, A., Cozzo, E., Kivelä, M., Moreno, Y., Porter, M. A., … Arenas, A. (2013). Mathematical formulation of multilayer networks. Physical Review X, 3(4), 041022. 10.1103/PhysRevX.3.041022

[bib32] de Vico Fallani, F., Richiardi, J., Chavez, M., & Achard, S. (2014). Graph analysis of functional brain networks: Practical issues in translational neuroscience. Philosophical Transactions of the Royal Society B: Biological Sciences, 369(1653), 20130521. https://doi.org/10.1098/rstb.2013.0521, 25180301 10.1098/rstb.2013.0521 PMC4150298 25180301

[bib33] Erdős, P., & Rényi, A. (1959). On random graphs. Publicationes Mathematicae, 6, 290–297.

[bib34] Faskowitz, J., Esfahlani, F. Z., Jo, Y., Sporns, O., & Betzel, R. F. (2020). Edge-centric functional network representations of human cerebral cortex reveal overlapping system-level architecture. Nature Neuroscience, 23(12), 1644–1654. https://doi.org/10.1038/s41593-020-00719-y, 33077948 33077948 10.1038/s41593-020-00719-y

[bib35] Fornito, A., & Bullmore, E. T. (2015). Reconciling abnormalities of brain network structure and function in schizophrenia. Current Opinion in Neurobiology, 30, 44–50. https://doi.org/10.1016/j.conb.2014.08.006, 25238608 25238608 10.1016/j.conb.2014.08.006

[bib36] Fornito, A., Zalesky, A., & Breakspear, M. (2015). The connectomics of brain disorders. Nature Reviews Neuroscience, 16(3), 159–172. https://doi.org/10.1038/nrn3901, 25697159 25697159 10.1038/nrn3901

[bib37] Fortunato, S. (2010). Community detection in graphs. Physics Reports, 486(3–5), 75–174. 10.1016/j.physrep.2009.11.002

[bib38] Fox, M. D., Snyder, A. Z., Vincent, J. L., Corbetta, M., Essen, D. C. V., & Raichle, M. E. (2005). From the cover: The human brain is intrinsically organized into dynamic, anticorrelated functional networks. Proceedings of the National Academy of Sciences, 102(27), 9673–9678. https://doi.org/10.1073/pnas.0504136102, 15976020 10.1073/pnas.0504136102 PMC1157105 15976020

[bib39] Friston, K., Kahan, J., Razi, A., Stephan, K. E., & Sporns, O. (2014). On nodes and modes in resting state fMRI. NeuroImage, 99, 533–547. https://doi.org/10.1016/j.neuroimage.2014.05.056, 24862075 24862075 10.1016/j.neuroimage.2014.05.056 PMC4121089

[bib40] Friston, K. J. (2009). Modalities, modes, and models in functional neuroimaging. Science, 326(5951), 399–403. https://doi.org/10.1126/science.1174521, 19833961 19833961 10.1126/science.1174521

[bib41] García-Pérez, G., Allard, A., Serrano, M. Á., & Boguñá, M. (2019). Mercator: Uncovering faithful hyperbolic embeddings of complex networks. New Journal of Physics, 21(12), 123033. 10.1088/1367-2630/ab57d2

[bib42] Ghavasieh, A., Bontorin, S., Artime, O., Verstraete, N., & De Domenico, M. (2021). Multiscale statistical physics of the pan-viral interactome unravels the systemic nature of SARS-CoV-2 infections. Communications Physics, 4(1). 10.1038/s42005-021-00582-8

[bib43] Ghavasieh, A., & Domenico, M. D. (2020). Enhancing transport properties in interconnected systems without altering their structure. Physical Review Research, 2(1). 10.1103/physrevresearch.2.013155

[bib44] Ghavasieh, A., Nicolini, C., & De Domenico, M. (2020). Statistical physics of complex information dynamics. Physical Review E, 102(5), 052304. https://doi.org/10.1103/PhysRevE.102.052304, 33327131 33327131 10.1103/PhysRevE.102.052304

[bib45] Goni, J., van den Heuvel, M. P., Avena-Koenigsberger, A., De Mendizabal, N. V., Betzel, R. F., Griffa, A., … Sporns, O. (2014). Resting-brain functional connectivity predicted by analytic measures of network communication. Proceedings of the National Academy of Sciences, 111(2), 833–838. https://doi.org/10.1073/pnas.1315529111, 24379387 10.1073/pnas.1315529111 PMC3896172 24379387

[bib46] Griffiths, K. R., Braund, T. A., Kohn, M. R., Clarke, S., Williams, L. M., & Korgaonkar, M. S. (2021). Structural brain network topology underpinning adhd and response to methylphenidate treatment. Translational Psychiatry, 11(1), 1–9. https://doi.org/10.1038/s41398-021-01278-x, 33654073 33654073 10.1038/s41398-021-01278-x PMC7925571

[bib47] Guillon, J., Attal, Y., Colliot, O., La Corte, V., Dubois, B., Schwartz, D., … Fallani, F. D. V. (2017). Loss of brain inter-frequency hubs in Alzheimer’s disease. Scientific Reports, 7(1), 1–13. https://doi.org/10.1038/s41598-017-07846-w, 28883408 28883408 10.1038/s41598-017-07846-w PMC5589939

[bib48] Guillon, J., Chavez, M., Battiston, F., Attal, Y., La Corte, V., Thiebaut de Schotten, M., … de Vico Fallani, F. (2019). Disrupted core-periphery structure of multimodal brain networks in Alzheimer’s disease. Network Neuroscience, 3(2), 635–652. https://doi.org/10.1162/netn_a_00087, 31157313 31157313 10.1162/netn_a_00087 PMC6542619

[bib49] Hahn, G., Ponce-Alvarez, A., Deco, G., Aertsen, A., & Kumar, A. (2019). Portraits of communication in neuronal networks. Nature Reviews Neuroscience, 20(2), 117–127. https://doi.org/10.1038/s41583-018-0094-0, 30552403 30552403 10.1038/s41583-018-0094-0

[bib50] Hernandez, L. M., Rudie, J. D., Green, S. A., Bookheimer, S., & Dapretto, M. (2015). Neural signatures of autism spectrum disorders: Insights into brain network dynamics. Neuropsychopharmacology, 40(1), 171–189. https://doi.org/10.1038/npp.2014.172, 25011468 25011468 10.1038/npp.2014.172 PMC4262896

[bib51] Holland, P. W., Laskey, K. V., & Leinhardt, S. (1983). Stochastic blockmodels: First steps. Social Networks, 5(2), 109–137. 10.1016/0378-8733(83)90021-7

[bib52] Honey, C. J., Kötter, R., Breakspear, M., & Sporns, O. (2007). Network structure of cerebral cortex shapes functional connectivity on multiple time scales. Proceedings of the National Academy of Sciences, 104(24), 10240–10245. https://doi.org/10.1073/pnas.0701519104, 17548818 10.1073/pnas.0701519104 PMC1891224 17548818

[bib53] Hwang, K., Hallquist, M. N., & Luna, B. (2012). The development of hub architecture in the human functional brain network. Cerebral Cortex, 23(10), 2380–2393. https://doi.org/10.1093/cercor/bhs227, 22875861 22875861 10.1093/cercor/bhs227 PMC3767958

[bib54] Lella, E., & Estrada, E. (2020). Communicability distance reveals hidden patterns of Alzheimer’s disease. Network Neuroscience, 4(4), 1007–1029. https://doi.org/10.1162/netn_a_00143, 33195946 33195946 10.1162/netn_a_00143 PMC7655045

[bib55] Liao, W., Ding, J., Marinazzo, D., Xu, Q., Wang, Z., Yuan, C., … Chen, H. (2011). Small-world directed networks in the human brain: Multivariate granger causality analysis of resting-state fMRI. NeuroImage, 54(4), 2683–2694. https://doi.org/10.1016/j.neuroimage.2010.11.007, 21073960 21073960 10.1016/j.neuroimage.2010.11.007

[bib56] Lin, S.-Y., Lin, C.-P., Hsieh, T.-J., Lin, C.-F., Chen, S.-H., Chao, Y.-P., … Kuo, L.-W. (2019). Multiparametric graph theoretical analysis reveals altered structural and functional network topology in Alzheimer’s disease. NeuroImage: Clinical, 22, 101680. https://doi.org/10.1016/j.nicl.2019.101680, 30710870 30710870 10.1016/j.nicl.2019.101680 PMC6357901

[bib57] Lohmann, G., Margulies, D. S., Horstmann, A., Pleger, B., Lepsien, J., Goldhahn, D., … Turner, R. (2010). Eigenvector centrality mapping for analyzing connectivity patterns in fMRI data of the human brain. PLoS ONE, 5(4), e10232. https://doi.org/10.1371/journal.pone.0010232, 20436911 20436911 10.1371/journal.pone.0010232 PMC2860504

[bib58] Maslov, S., & Sneppen, K. (2002). Specificity and stability in topology of protein networks. Science, 296(5569), 910–913. https://doi.org/10.1126/science.1065103, 11988575 11988575 10.1126/science.1065103

[bib59] Masuda, N., Porter, M. A., & Lambiotte, R. (2017). Random walks and diffusion on networks. Physics Reports, 716–717, 1–58. 10.1016/j.physrep.2017.07.007

[bib60] Meunier, D., Lambiotte, R., Fornito, A., Ersche, K., & Bullmore, E. T. (2009). Hierarchical modularity in human brain functional networks. Frontiers in Neuroinformatics, 3, 37. https://doi.org/10.3389/neuro.11.037.2009, 19949480 19949480 10.3389/neuro.11.037.2009 PMC2784301

[bib61] Muldoon, S. F., Bridgeford, E. W., & Bassett, D. S. (2016). Small-world propensity and weighted brain networks. Scientific Reports, 6(1). https://doi.org/10.1038/srep22057, 26912196 10.1038/srep22057 PMC4766852 26912196

[bib62] Network Geometry - Mercator. (2020). GitHub, https://github.com/networkgeometry/mercator.

[bib63] Newman, M. E. J. (2017). Networks an introduction. Oxford: Oxford University Press. 10.1093/oso/9780198805090.003.0001

[bib64] Noh, J. D., & Rieger, H. (2004). Random walks on complex networks. Physical Review Letters, 92(11), 118701. https://doi.org/10.1103/PhysRevLett.92.118701, 15089179 15089179 10.1103/PhysRevLett.92.118701

[bib65] Nooner, K., Colcombe, S., Tobe, R., Mennes, M., Benedict, M., Moreno, A., … Milham, M. (2012). The NKI-Rockland sample: A model for accelerating the pace of discovery science in psychiatry. Frontiers in Neuroscience, 6, 152. https://doi.org/10.3389/fnins.2012.00152, 23087608 23087608 10.3389/fnins.2012.00152 PMC3472598

[bib66] Papadopoulos, F., Kitsak, M., Serrano, M. Á., Boguñá, M., & Krioukov, D. (2012). Popularity versus similarity in growing networks. Nature, 489, 537–540. https://doi.org/10.1038/nature11459, 22972194 22972194 10.1038/nature11459

[bib67] Papo, D., Buldú, J. M., Boccaletti, S., & Bullmore, E. T. (2014). Complex network theory and the brain. Philosophical Transactions of the Royal Society B: Biological Sciences, 369(1653), 20130520. https://doi.org/10.1098/rstb.2013.0520, 25180300 10.1098/rstb.2013.0520 PMC4150297 25180300

[bib68] Peixoto, T. P. (2019). Bayesian stochastic blockmodeling. In Advances in network clustering and blockmodeling (pp. 289–332). Newark, NJ: John Wiley & Sons. 10.1002/9781119483298.ch11

[bib69] Penny, W., Iglesias-Fuster, J., Quiroz, Y. T., Lopera, F. J., & Bobes, M. A. (2018). Dynamic causal modeling of preclinical autosomal-dominant Alzheimer’s disease. Journal of Alzheimer’s Disease, 65(3), 697–711. https://doi.org/10.3233/JAD-170405, 29562504 10.3233/JAD-170405 PMC6923812 29562504

[bib70] Qiu, M.-G., Ye, Z., Li, Q.-Y., Liu, G.-J., Xie, B., & Wang, J. (2011). Changes of brain structure and function in ADHD children. Brain Topography, 24(3–4), 243–252. https://doi.org/10.1007/s10548-010-0168-4, 21191807 21191807 10.1007/s10548-010-0168-4

[bib71] Raimondo, S., & Domenico, M. D. (2021). Measuring topological descriptors of complex networks under uncertainty. Physical Review E, 103(2). https://doi.org/10.1103/PhysRevE.103.022311, 33735966 10.1103/PhysRevE.103.022311 33735966

[bib72] Reuter-Lorenz, P. A., & Cappell, K. A. (2008). Neurocognitive aging and the compensation hypothesis. Current Directions in Psychological Science, 17(3), 177–182. 10.1111/j.1467-8721.2008.00570.x

[bib73] Rubinov, M., & Bullmore, E. (2013). Schizophrenia and abnormal brain network hubs. Dialogues in Clinical Neuroscience, 15(3), 339–349. https://doi.org/10.31887/DCNS.2013.15.3/mrubinov, 24174905 24174905 10.31887/DCNS.2013.15.3/mrubinov PMC3811105

[bib74] Rudie, J. D., Brown, J., Beck-Pancer, D., Hernandez, L., Dennis, E., Thompson, P., … Dapretto, M. (2013). Altered functional and structural brain network organization in autism. NeuroImage: Clinical, 2, 79–94. https://doi.org/10.1016/j.nicl.2012.11.006, 24179761 10.1016/j.nicl.2012.11.006 PMC3777708 24179761

[bib75] Schirner, M., McIntosh, A. R., Jirsa, V., Deco, G., & Ritter, P. (2018). Inferring multi-scale neural mechanisms with brain network modelling. eLife, 7. https://doi.org/10.7554/elife.28927, 29308767 10.7554/eLife.28927 PMC5802851 29308767

[bib76] Seguin, C., van den Heuvel, M. P., & Zalesky, A. (2018). Navigation of brain networks. Proceedings of the National Academy of Sciences, 115(24), 6297–6302. https://doi.org/10.1073/pnas.1801351115, 29848631 10.1073/pnas.1801351115 PMC6004443 29848631

[bib77] Sellke, T., Bayarri, M., & Berger, J. O. (2001). Calibration of *ρ* values for testing precise null hypotheses. The American Statistician, 55(1), 62–71. 10.1198/000313001300339950

[bib78] Sheng, X., Chen, H., Shao, P., Qin, R., Zhao, H., Xu, Y., & Bai, F. (2021). Brain structural network compensation is associated with cognitive impairment and Alzheimer’s disease pathology. Frontiers in Neuroscience, 15(February), 1–13. https://doi.org/10.3389/fnins.2021.630278, 33716654 10.3389/fnins.2021.630278 PMC7947929 33716654

[bib79] Sporns, O., & Betzel, R. F. (2016). Modular brain networks. Annual Review of Psychology, 67, 613–640. https://doi.org/10.1146/annurev-psych-122414-033634, 26393868 10.1146/annurev-psych-122414-033634 PMC4782188 26393868

[bib80] Sporns, O., Chialvo, D. R., Kaiser, M., & Hilgetag, C. C. (2004). Organization, development and function of complex brain networks. Trends in Cognitive Sciences, 8(9), 418–425. https://doi.org/10.1016/j.tics.2004.07.008, 15350243 15350243 10.1016/j.tics.2004.07.008

[bib81] Su, H., Chen, D., Pan, G.-J., & Zeng, Z. (2021). Identification of network topology variations based on spectral entropy. IEEE Transactions on Cybernetics, 1–11. https://doi.org/10.1109/TCYB.2021.3070080, 33878010 10.1109/TCYB.2021.3070080 33878010

[bib82] Tzourio-Mazoyer, N., Landeau, B., Papathanassiou, D., Crivello, F., Etard, O., Delcroix, N., … Joliot, M. (2002). Automated anatomical labeling of activations in SPM using a macroscopic anatomical parcellation of the MNI MRI single-subject brain. NeuroImage, 15(1), 273–289. https://doi.org/10.1006/nimg.2001.0978, 11771995 11771995 10.1006/nimg.2001.0978

[bib83] van den Heuvel, M. P., & Pol, H. E. H. (2010). Exploring the brain network: A review on resting-state fMRI functional connectivity. European Neuropsychopharmacology, 20(8), 519–534. https://doi.org/10.1016/j.euroneuro.2010.03.008, 20471808 20471808 10.1016/j.euroneuro.2010.03.008

[bib84] van den Heuvel, M. P., & Sporns, O. (2013). Network hubs in the human brain. Trends in Cognitive Sciences, 17(12), 683–696. https://doi.org/10.1016/j.tics.2013.09.012, 24231140 24231140 10.1016/j.tics.2013.09.012

[bib85] Vértes, P. E., Alexander-Bloch, A. F., Gogtay, N., Giedd, J. N., Rapoport, J. L., & Bullmore, E. T. (2012). Simple models of human brain functional networks. Proceedings of the National Academy of Sciences, 109(15), 5868–5873. https://doi.org/10.1073/pnas.1111738109, 22467830 10.1073/pnas.1111738109 PMC3326510 22467830

[bib86] Watts, D. J., & Strogatz, S. H. (1998). Collective dynamics of ‘small-world’ networks. Nature, 393(6684), 440–442. https://doi.org/10.1038/30918, 9623998 9623998 10.1038/30918

[bib87] Williamson, B. J., De Domenico, M., & Kadis, D. S. (2021). Multilayer connector hub mapping reveals key brain regions supporting expressive language. Brain Connectivity, 11(1), 45–55. https://doi.org/10.1089/brain.2020.0776, 33317399 33317399 10.1089/brain.2020.0776 PMC7891212

[bib88] Yamamoto, H., Moriya, S., Ide, K., Hayakawa, T., Akima, H., Sato, S., … Hirano-Iwata, A. (2018). Impact of modular organization on dynamical richness in cortical networks. Science Advances, 4(11), eaau4914. https://doi.org/10.1126/sciadv.aau4914, 30443598 30443598 10.1126/sciadv.aau4914 PMC6235526

[bib89] Yao, Z., Zhang, Y., Lin, L., Zhou, Y., Xu, C., & Jiang, T. (2010). Abnormal cortical networks in mild cognitive impairment and Alzheimer’s disease. PLoS Computational Biology, 6(11). https://doi.org/10.1371/journal.pcbi.1001006, 21124954 10.1371/journal.pcbi.1001006 PMC2987916 21124954

[bib90] Zalesky, A., Fornito, A., & Bullmore, E. T. (2010). Network-based statistic: Identifying differences in brain networks. NeuroImage, 53(4), 1197–1207. https://doi.org/10.1016/j.neuroImage.2010.06.041, 20600983 20600983 10.1016/j.neuroimage.2010.06.041

[bib91] Zheng, M., Allard, A., Hagmann, P., Alemán-Gómez, Y., & Serrano, M. Á. (2020). Geometric renormalization unravels self-similarity of the multiscale human connectome. Proceedings of the National Academy of Sciences, 117(33), 20244–20253. https://doi.org/10.1073/pnas.1922248117, 32759211 10.1073/pnas.1922248117 PMC7443937 32759211

